# The fuzzy Kullback–Leibler divergence for estimating parameters of the probability distribution in fuzzy data: an application to classifying Vietnamese Herb Leaves

**DOI:** 10.1038/s41598-023-40992-y

**Published:** 2023-09-04

**Authors:** Hoa Le, Vu Ngoc Thanh Sang, Le Nhi Lam Thuy, Pham The Bao

**Affiliations:** 1 University of Economics and Law and Vietnam National University, Ho Chi Minh City, Vietnam; 2 Sai Gon University, Ho Chi Minh city, Vietnam

**Keywords:** Classification and taxonomy, Image processing, Machine learning

## Abstract

In this paper, we address the challenge of estimating probability distributions which are typically represented by parameter-based values. However, this estimation is prone to errors and does not comprehensively capture the nature of real-world data. Additionally, real-world data often follows a mixed form of probability distributions, where sub-datasets may contain incomplete information. To enhance flexibility, especially in classification problems, we propose a new method for describing parameters estimated through Bayesian statistics. Our method introduces fuzzy parameters and assesses the similarity between probability distributions using the fuzzy extended Kullback–Leibler divergence. We demonstrate the practical application of our approach in Vietnamese Herb Leaves classification. By incorporating fuzzy parameters and leveraging Bayesian statistics, our method provides more robust estimations of probability distributions and enables improved flexibility in classification tasks.

## Introduction

Handling practical data as a single distribution presents inherent challenges and is deemed inappropriate. To overcome this limitation, a promising solution emerges: modeling the probability distribution of the given data as a mixture of probability distributions. Accomplishing this involves determining the correct forms of probability distributions for mixed data, a task achieved through the utilization of the Expectation–Maximization (EM) algorithm^1,2^. This algorithm plays a crucial role in accurately estimating the parameters and components of the mixed model, enabling a more comprehensive representation of the underlying data structure.

In the context of the mixture model, a crucial step involves determining the number of components and parameters required for the mixed model. The Gaussian mixture model (GMM), a widely employed form consisting of a mixture of normal distributions, serves as a common starting point. To accurately identify these components and parameters, various criteria are employed, such as the Bayesian information criterion (BIC) of Schwarz, Akaike’s information criterion (AIC), normalized entropy criterion, or cross-validation^3^. Regardless of the criterion used, it is imperative to select the appropriate number of components, denoted as $g_0$ , in a manner that minimizes the discrepancy between the experimental data and the mixed model of normal probability distributions. Decisions regarding the mixture of component probability distributions are made based on diverse algorithms^1^ or hypothesis testing, typically denoted as $H_0:g=g_0.$

The parameters of a mixed model are typically estimated empirically. The estimation process concludes when the errors of the estimate become sufficiently small, with values less than $\varphi > 0$. The resulting estimated parameter, denoted as ${\hat{\theta }}$, is then utilized for further inferences. In practice, the possible parameter values revolve around the estimated value ${\hat{\theta }}$, indicating a range of $\theta \in ({\hat{\theta }} - \varepsilon , {\hat{\theta }} + \varepsilon )$. The estimated results pertaining to the model’s parameters, encompassing both Bayesian and non-Bayesian statistics, are presented in relation to fuzzy sets^4^. Consequently, this estimation process justifies considering the parameters within the estimation models as fuzzy numbers.

Furthermore, it is important to acknowledge that a significant portion of the collected data should be treated as fuzzy numbers^5^. Numerous studies have employed different approaches to estimate the probability distribution of fuzzy data. These methods include utilizing a Gaussian mixture model of fuzzy numerical values^6^, a fuzzy Gaussian mixture model (FGMM) based on the dissimilarity of fuzzy C-means^7^, a similarity measure between fuzzy sets through entropy^8^, or hesitant ordered weighted similarity measures^9^. However, simply counting the number of observations of the fuzzy number intersecting a $\delta$-cut and the number of observations of the fuzzy number union with a $\delta$-cut is insufficient to determine the entropy between two fuzzy sets. Therefore, it remains necessary to investigate the similarity between fuzzy probability distributions or probability distributions with fuzzy parameters.

The primary objective of this research was to address the limitations associated with parameter estimation in probability distributions and develop an alternative approach that enhances flexibility and adaptability in statistical models, particularly within classification problems. The novelty of this study is the recognizing that probability distributions are typically parameter-based. This approach emphasizes the need for a fresh perspective on parameter estimation of the mixed form of probability distributions commonly observed in real-world data and the importance of considering the complexity and diversity of data patterns.

This research study makes valuable contributions to the field by proposing a novel method that incorporates parameters into statistical models, leading to enhanced flexibility and adaptability. Furthermore, the study applied the fuzzy extended Kullback–Leibler divergence for parameter description and comparison between probability distributions. By demonstrating the effectiveness of this approach and providing tangible evidence of its potential applications and benefits, particularly in the context of classifying Vietnamese Herb leaves, the research showcases its potential and application in real-world scenarios.

## Literature review

The novel fuzzy probability density function, introduced in this research, represents a relatively less explored area in the field, as evidenced by the limited number of studies focusing on this concept. The current research primarily concentrates on applying this new fuzzy probability density function to a single probability distribution, marking a significant advancement in the field. However, it’s crucial to note that this focus is limited to single probability distributions. Furthermore, the concept of a mixture of fuzzy probability density functions, capable of handling real-world data following a mixture of probability distributions, emerges as a novel and promising area of research. Despite its potential to address the limitations of existing methods, particularly when dealing with skewed distributions, this concept has not yet been extensively studied, underscoring the need for further research in this area as presented in the Table 1.

**Table 1:** Related works for various distribution models and their key contributions and limitations.

Refs.	Distribution		Paramter		Key contribution	Limitations
	Name	Kind	Name	Kind		
^10^	Normal distribution	Deterministic	Neutrosophic mean and standard deviation	Fuzzy	Used for testing multiple neutrosophic population means simultaneously	Employed within an uncertain context
^11^	Normal distribution	Deterministic	Mean and standard deviation	Deterministic	Utilization of real-valued random variable representations with unknown distributions following the central limit theorem	Assumption of independence in real-world scenarios and inability to handle skewed data in certain cases
^12^	Uniform distribution	Deterministic	Lower limit and upper limit	Deterministic	Application of uniform distribution in random number generation and problems with equally likely outcomes	It assumes equal likelihood for all outcomes and lacks the ability to model skewed data
^13^	Exponential distribution	Deterministic	Rate parameter	Deterministic	Exponential distribution modeling of time between events in Poisson point processes	Based on the assumption of constant event rates, which may not always reflect reality
^14^	Poisson Distribution	Deterministic	Rate parameter	Deterministic	Modeling the number of event occurrences in time or space	Based on the assumption of constant event rates
^15^	Local Histogram	Deterministic	Bin size	Deterministic	Simple to construct and interpret, requiring minimal mathematical calculations	Sensitivity to bin size and potential bias in representing underlying data distribution
^16^	GARCH Model	Deterministic	Mean, variance, lag order for past returns, lag order for past conditional volatility, GARCH model parameters	Deterministic	Suitability for linguistic interpretation of density changes	GARCH Model’s local maxima issues during parameter estimation.
^17^	Fuzzy GARCH Model	Fuzzy	Mean for each rule, membership function parameters	Fuzzy	Interpreting gradual changes in output density, capture fat tails, and multimodality	Potential local optima issues and estimation challenges in highly parametrized Fuzzy GARCH models
^18^	Fuzzy Coefficient Volatility (FCV) models	Fuzzy	Parameters modeled through defuzzification or linear fuzzy numbers	Fuzzy	CV models’ intuitive handling of parameter uncertainty and superior fuzzy forecasts	Limitation of linear membership functions in accurately representing linguistic terms and decision-making difficulties in their use

Neutrosophic statistics and fuzzy logic are distinct statistical approaches that have evolved from classical binary logic to represent and quantify uncertainty and vagueness in data and knowledge bases^10^. These methodologies have gained substantial attention due to their ability to handle imprecise and uncertain information. Neutrosophic statistics is grounded in the principles of neutrosophic logic, which does incorporate a measure of indeterminacy. They enhance the ’degrees of truth’ concept by accommodating indeterminacy, proving beneficial when working with incomplete or conflicting data^19^. On the other hand, fuzzy statistics do not factor in the measure of indeterminacy. Fuzzy logic embraces the concept of ’partial truth’, effectively dealing with data’s imprecision and vagueness, proving its worth in numerous domains like AI, machine learning, and control systems. The emphasis of this research is based on fuzzy logic because of its widespread adoption and comprehensible structure. While neutrosophic logic has been investigated to a degree, it remains an area with significant potential and is earmarked as a promising field for upcoming studies and applications.

The estimations of fuzzy probability density functions based on parametric or non-parametric methods as based on histogram method^20^, empirical cumulative distribution function-based method, kernel method^5,21^. The histogram method in determining the probability density function is applicable with additional smoothing techniques. In addition, the empirical cumulative distribution function-based method is determined based on the lower bound probability density function $f^L_\delta$ and the upper bound probability density function $f^U_\delta$, such that the expectations of these functions converge to the exact probability density function *f*, and the variance converges to 0. Moreover, suppose the smoothing function is guaranteed, the bias of the lower bound probability density function $f^L_\delta$ and the upper bound probability density function $f^U_\delta$ converge to 0, and the variance converges to zero. By employing these estimation methods and optimizing their smoothing techniques, researchers can obtain reliable and precise approximations of fuzzy probability density functions, thereby enabling more accurate modeling and analysis of uncertain phenomena.

In addition, the kernel method effectively estimates the overall form and shape of fuzzy probability density functions, excelling at capturing their complex structure^22^. However, it lacks the capability to identify the specific mixture of probability distributions that contribute to the formation of the fuzzy density function. Consequently, when using Bayesian statistics alone, the kernel method cannot determine the corresponding parameters of the underlying probability distributions. In practical applications, incorporating new data and utilizing the kernel method to determine the form of a new fuzzy probability density function necessitates restarting the entire estimation process. It is unable to leverage previously acquired knowledge or progress, requiring a fresh start each time. Conversely, if the mixture form of the underlying probability distributions is already known or pre-defined, inference techniques can accurately determine the parameters and provide precise information^23^. This prior knowledge of the mixture form streamlines the estimation process, saving valuable time and computational resources.

Alternatively, the fuzzy probability density function or the conditional fuzzy probability density function can be determined utilizing the Generalized Autoregressive Conditional Heteroskedasticity (GARCH) model^17,24^. The GARCH model bases its accuracy on an average calculation of variables, aiming for the minimum variance. These methods’ advantages are their independence from any preset probability distribution form of the data, allowing the results to derive purely from the data itself. If we collect data from the same object but at a different time, the probability density functions need to be recalculated. Similarly, fuzzy probabilities are calculated using the quantile regression model at specific levels^25^. To enhance the accuracy of these calculations, we should use more exact measuring tools such as entropy, relative entropy, or Kullback–Leibler divergence. These tools are helpful as they clarify how the distribution changes and measure the difference between various probability density functions.

Feature parameters of fuzzy data are also crucial for evaluating the characteristics of fuzzy data, such as expectation, variance, covariance, higher-order moments, and entropy^26,27^. Notably, the Kullback–Leibler divergence, which is an effective tool for measuring the difference between two probability distributions, is not usually applied in fuzzy data contexts. This under-utilization hints at a research gap, one that could potentially expand the comprehension and utilization of fuzzy data analysis upon exploration.

The fuzzy probability density function is considered as a density curve that resides in two $\alpha -$level functions^28^. Estimating the parameters of the probability density function requires numerous trials to confirm the appropriateness of the estimated values. In real-world situations, it is frequently observed that a combination of various probability distributions fits better than just a singular one. Therefore, ensuring a good alignment between parameters and the projected probability distributions is essential. This area stands as a critical focus for more detailed study and research.

Regarding the simulation study, our research team has conducted an extensive analysis in a separate research paper^28^. Our previous study primarily concentrated on single probability distributions, neglecting the exploration of the mixture form. We proposed an algorithm that encompasses both simulated and real datasets, explicitly targeting the analysis of stock price data. In that study, we estimated fuzzy probability density functions using a mixture of probability distributions located within the region between the $\delta -$level functions. During our previous examination, fuzzy probability density functions were emphasized primarily on single probability distributions, neglecting the exploration of the mixture form.

Under these circumstances, in this research, we propose a novel approach that treats the parameters of probability distributions as fuzzy numbers, drawing inspiration from the parameter-centric probability distribution perspective of Bayesian statistics. Making assumptions about these parameters becomes crucial when dealing with a mixture of probability distributions. Under such conditions, the component probability distributions, formulated based on the data, only capture a piece of the total information. We also introduce a new theorem to determine the fuzzy equivalence between two fuzzy probability distributions. The theory is accomplished using the fuzzy adaptation of the Kullback–Leibler divergence, which is extensively used in various practical fields.

## Parameter estimation in Gaussian mixture model

We make the assumption that observations $x_1,x_2,\ldots ,x_n$ are independent and conform to the same probability distribution. Here, $z_{ig}$ is used to denote component membership. Specifically, $z_{ig}=1$ indicates that observation *i* is a member of component *g*, while $z_{ig}=0$ implies otherwise. The likelihood function can be expressed by formula (1) as presented in^29^:

1 $$\begin{aligned} L_C (v)=\prod _{i=1}^n \prod _{g=1}^G [\pi _g \Phi (x_i | \mu _g,\sigma _g^2 )]^{z_{ig}}, \end{aligned}$$

where *v* represents the model parameters, which can be detailed as $v=(\pi _1,\ldots ,\pi _G;\mu _1,\ldots ,\mu _G; \sigma _1^2,\ldots ,\sigma _G^2).$ By applying the natural logarithm to Eq. (1), we obtain the complete-data log-likelihood which is represented by Eq. (2) as mentioned in^29^:

2 $$\begin{aligned} l_C (v)=\sum _{i=1}^n \sum _{g=1}^G z_{ig} [\log \pi _g +\log \Phi (x_i | \mu _g,\sigma _g^2)]. \end{aligned}$$

In the E-step, $z_{ig}$ are replaced by their expected value in formula (3)^29^:

3 $$\begin{aligned} {\hat{z}}_{ig}=\frac{{\hat{\pi }}_g \Phi (x_i |{\hat{\mu }}_g,{\hat{\sigma }}_g^2)}{\sum _{g=1}^G {\hat{\pi }}_g \Phi (x_i |{\hat{\mu }}_g,{\hat{\sigma }}_g^2)}, \end{aligned}$$

where $$i=1,2,\ldots ,n$$ and $$g=1,2,\ldots ,G$$.

The model parameters that maximize the anticipated value of the complete-data log-likelihood can be achieved in M-step using the formula (4)^29^:

4 $$\begin{aligned} {\hat{\pi }}_g=\frac{n_g}{n},{\hat{\mu }}_g= \frac{\sum _{i=1}^n {\hat{z}}_{ig} x_i}{n_g}, {\hat{\sigma }}_g^2=\frac{\sum _{i=1}^n {\hat{z}}_{ig} (x_i-{\hat{\mu }}_g)^2)}{n_g-1}. \end{aligned}$$

**Figure 1:**
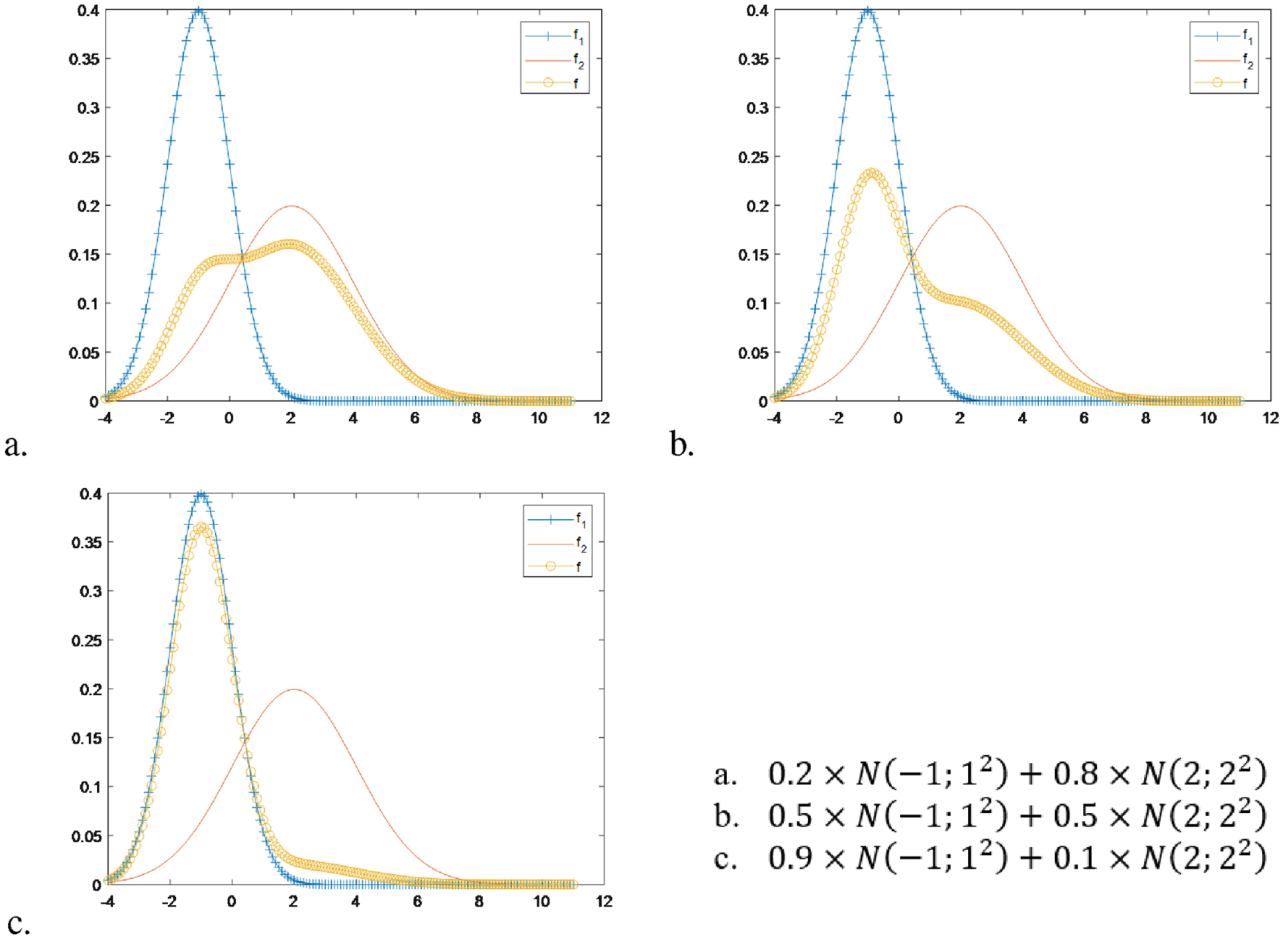
The mixture of two normal distributions with the component density functions as $f_1 \sim N(-1;1^2)$ and $f_2 \sim N(2;2^2)$. The mixed density function, denoted as *f*, represents the combined distribution resulting from the mixture of these two components.

**Figure 2:**
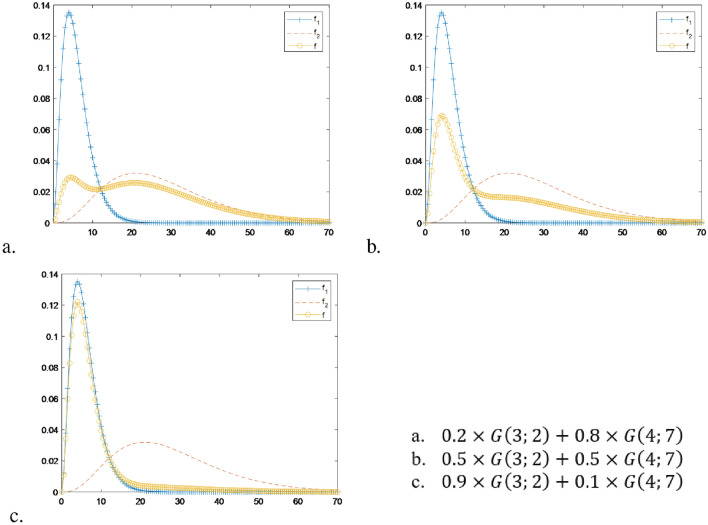
The mixture of two gamma distributions in which the component density functions are $f_1\sim G(3;2)$ and $f_2 \sim G(4;7)$ and the mixed density function is *f*.

Despite the utility of the EM algorithm, it does come with several drawbacks, which are as follows:

- The first limitation is that it is required to pre-define the number of components in the mixture of probability distributions. Additionally, we have to choose the optimal number of mixtures that best represent the data based on model selection. Indeed, Fig. 1 shows a mixture of two normal distributions $N(\mu ,\sigma ^2)$ are $N(-1;1^2)$ and $N(2;2^2)$. Changes in their rates influence the overall shape of the mixture. As observed in Fig. 1a, the mixed distribution *f* clearly exhibits a two-peak structure. However, when scaling the component distributions with an equal proportion of 50 % for each distribution (Fig. 1b), we observe that the probability density function displays two indistinct peaks. Notably, when the probability ratio changes to 90 and 10% in Fig. 1c, the previously well-defined probability distribution becomes unimodal, characterized by a single peak. In such scenarios, it is appropriate to approximate the distribution as a single probability distribution, such as a normal distribution or a skewed distribution.
- Secondly, when it comes to estimating the component probability distributions of a mixture of probability distributions, the EM algorithm is typically applicable only to mixtures of normal distributions. However, attempting to approximate the data using a mixed form of normal distributions does not ensure complete accuracy, as the two component distributions are skewed and have heavy tails. To achieve a more precise representation, it is necessary to consider mixtures of skewed distributions that can capture the characteristics of the data more effectively. In Fig. 2, we observe a mixture of two gamma distributions, namely $G(k;\theta )$, where *k* and $\theta$ represent the shape and scale parameters. These parameters determine the rates of change within the distributions. The two-component gamma distributions in question are *G*(3; 2) and *G*(4; 7), which possess distinct shapes due to their differing ratios. Upon examining Fig. 2a, we observe two peaks, whereas Fig. 2b and c display a single peak. The heavy distribution in the right tail of Fig. 2a suggests that it should be approximated as a mixture of two normal distributions. This estimation differs from that of Fig. 2b and c. Hence, in order to achieve an accurate approximation of Fig. 2b and c, it is necessary to employ a minimum of one mixture comprising three normal distributions, even though the actual distribution is a mixture of only two gamma distributions.
  The exponential distribution Exp($\lambda$) with rate parameter $\lambda$ has an expected value of $\frac{1}{\lambda }$ and a variance of $\frac{1}{{\lambda }^2}$. It is considered a special case of the gamma distribution, which is a two-parameter family of continuous probability distributions. In the gamma distribution with shape parameter $k>0$ and scale parameter $\theta >0$, the mean is $k\theta$, and the variance is $k{\theta }^2$. When $k=1$, the gamma distribution becomes the exponential distribution. Therefore, the gamma distribution encompasses the exponential distribution. Additionally, the gamma distribution and mixtures of gamma distributions can encompass probability distributions that only take positive values, normal distributions, and mixtures of normal distributions, which can include both positive and negative values.

Although, it is possible to calculate the parameters of the normal distribution, the problem arises when the probability distributions are not normal. This problem leads to either incorrect approximation across the mixture of normal distributions or computationally intensive. Therefore, we need to determine the most appropriate probability distribution format for component probability distributions, based on mixed distribution information. Thus, we infer the parameters as well as the ratios of the respective component probability distributions.

Calculating the parameters of the normal distribution is feasible. However, complications arise when dealing with probability distributions that deviate from normality. In such cases, approximating the mixture using a combination of normal distributions may result in incorrect representations or demand computationally intensive procedures. Hence, it becomes crucial to identify the most suitable probability distribution format for the component probability distributions based on the information provided by the mixed distribution. Consequently, we infer both the parameters and ratios of the corresponding component probability distributions to ensure an appropriate approximation.

## Estimate the parameters of the component probability distribution in the mixed model

Given that the dataset is divided into sub-datasets, the subsequent challenge is to identify the most suitable single probability distribution for each sub-dataset. It is essential to recognize that these sub-datasets represent incomplete component data, as there is still potential for data misclassification. Consequently, the estimation of probability distributions for the sub-datasets differs from that of the complete dataset, taking into account the potential inaccuracies arising from incomplete information.

### Estimate the parameters of the component probability distribution

We introduce a novel algorithm designed specifically for estimating the parameters within the component probability distribution of sub-datasets.
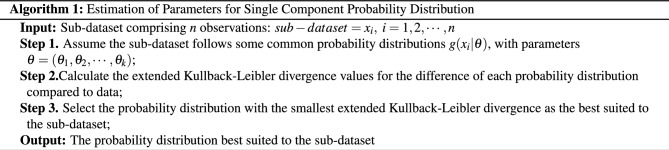


The algorithm presented in Algorithm 1 aims to estimate the parameters for a single component probability distribution based on a given sub-dataset. In step 1, the sub-dataset, consisting of *n* observations, is provided as input: $sub-dataset =x_i, \ i=1,2,\ldots ,n$. It is assumed that the sub-dataset follows some common probability distributions $g(x_i| \theta )$, where $\theta =(\theta _1,\theta _2,\ldots ,\theta _k)$ represents the parameters of the distributions. For instance, in the case of a normal distribution, which requires two parameters—the mean and the standard deviation—the sample mean and the standard deviation are used as the estimation for these parameters. Under these circumstances, the sample mean serves as an estimate for the mean of the target distribution, while the sample standard deviation provides an estimate for the standard deviation of the target distribution. In the step 2, the extended Kullback–Leibler divergence values are calculated to measure the difference between each probability distribution and the actual data. This step helps quantify the fit of each distribution to the sub-dataset. Finally, in step 3, the probability distribution with the smallest extended Kullback–Leibler divergence is selected as the most suitable distribution for the given sub-dataset. This selection is based on the notion that the distribution with the lowest divergence indicates a better fit to the observed data. The output of the algorithm is the probability distribution that is considered best suited to the sub-dataset. By following these steps, the algorithm facilitates the estimation of parameters for a single component probability distribution, aiding in the identification of the most appropriate distribution that aligns with the given sub-dataset.

The Kullback–Leibler divergence between two probability density functions *f*(*x*) and *g*(*x*) is determined by the formula (5)^30^:

5 $$\begin{aligned} D(f||g)=\int f(x) \log \frac{f(x)}{g(x)} dx. \end{aligned}$$

We use the extended Kullback–Leibler divergence be determined by the formula (6)^31^:

6 $$\begin{aligned} KLD(f||f_{emp}) \approx \sum _{j=1}^n Prob_f ((x_{j -1};x_j ])\times \log \frac{Prob_f ((x_{j -1};x_j ])}{Prob_{f_{emp}} ((x_{j -1};x_j ])}, \end{aligned}$$

in which $f_{emp}$ is the empirical probability of data. In this context, it is important to note that the samples $x_{1}, x_{2},\ldots , x_{n}$ are arranged in ascending order, as indicated by the notation $(x_{j-1}; x_{j}]$. These represent non-overlapping consecutive intervals. The ordering of samples is a common requirement in certain statistical methods, particularly those that involve cumulative probabilities or the calculation of specific types of divergence or distance measures. In the case of Eq. (6), this sorted order of samples is utilized to compute the extended Kullback–Leibler divergence. This divergence quantifies the difference between the empirical probability distribution of the data and the theoretical probability distribution being fitted to the data.

The extended Kullback–Leibler divergence is a more effective measure compared to metrics such as mean square error (MSE), mean error (ME), and mean absolute error (MAE) when it comes to determining the appropriate probability distribution for a single set of data.

### Estimated component parameters based on Bayesian statistics

The parameters of the estimating component probability distribution are based on incomplete data, which introduces uncertainty in determining the correct probability distribution. As a result, the proposed Algorithm 2 offers improved accuracy in estimating the parameters to mitigate the challenges posed by incomplete data.
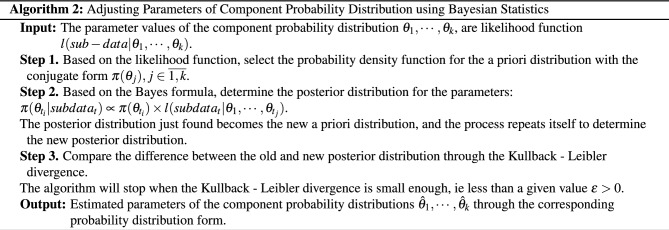


The adjusted probability density function in Algorithm 2 indeed represents the posterior probability density function. The parameters of this function are calculated based on the product of non-negative functions, ensuring that they themselves are non-negative. In line with the formula in Bayesian statistics, this non-negative function is divided by a constant, specifically the integral of the non-negative function over the entire defined domain. Consequently, the newly calculated function satisfies the properties of a probability density function: it is non-negative and its integral over its defined domain is exactly equal to 1.

Moreover, we have constructed a dataset known as the Vietnamese Herb Leaf Images (V-Herb) database. This database was created by collecting photographs of medicinal plants, which were subsequently identified by herbalists and botanists. The number of photographs obtained for each species or strain varied based on specific requirements. The leaf images in the database encompass both Vietnamese herbal plants and general leaves, exhibiting diverse morphological and structural characteristics influenced by factors such as region, climate, and soil conditions. The V-Herb database comprises a total of 3807 distinct leaf images corresponding to 296 unique Vietnamese herbal species. Table 2 showcases some examples of Vietnamese herbal species present in our V-Herb database. It is worth noting that all the color images were captured using mobile devices, against different backgrounds, at various times, and under varying lighting conditions.

**Table 2:** Examples of Vietnamese herbal species in our V-Herb database.

Vietnamese herbal species	Scientific names	Vietnamese herbal species	Scientific name
Ắc ó	*Acanthus integrifolius*	Muồng trâu	*Senna alata*
Bạch hoa xà	*Plumbago zeylanica*	Quỳnh lam	*Gonocaryum lobbianum*
Cà hai lá	*Solanum diphyllum*	Ngọc nữ biển	*Clerodendrum inerme* (L.) Gaertn
Đinh lăng lá rỗ	*Polyscias guilfoylei*	Sâm cau lá lớn	*Curculigo capitulata* (Lour.) Kuntze
Hoắc hương	*Pogostemon cablin*	Sử quân tử	*Combretum indicum*
Hương nhu trắng	*Ocimum gratissimum*	Thông thiên	*Thevetia peruviana*
Lược vàng	*Callisia fragrans*	Xảo tam phân	*Paramignya trimera*

The features from the leaf images were extracted utilizing two methods: histogram of oriented gradients (HOG) and local binary pattern (LBP). These techniques allow for the capture of distinct characteristics and patterns present in the leaf images.

The original objective of the HOG was to detect humans in cluttered backgrounds by quantifying gradient orientation occurrences. The working principle of HOG involves dividing the image into cells and calculating a histogram of gradient orientations within each cell. The computation of gradients is performed in both the *x* and *y* directions, capturing the magnitude and orientation of the gradients using the following formula (7)^32^:

7 $$\begin{aligned} G_x(x,y)= I(x+1,y)- I(x-1,y); G_y(x,y)= I(x,y+1)- I(x,y-1), \end{aligned}$$

where *I*(*x*, *y*) represents the intensity of the pixel at coordinates (*x*, *y*). For each pixel, the gradient value *G*(*x*, *y*) and gradient direction $\theta (x,y)$ are calculated from two directions determined by (8) and (9) fomulars.

8 $$\begin{aligned} G(x,y)= & {} \sqrt{G_x^2 (x,y) +G_y^2 (x,y)},\end{aligned}$$

9 $$\begin{aligned} \theta (x,y)= & {} \tan ^{-1} \frac{G_y (x,y)}{G_x (x,y)}. \end{aligned}$$

Following the computation of gradients, each pixel within a cell contributes a weighted vote to an orientation-based histogram. This histogram divides the range of gradient angles into k bins. Normalizing the histograms of all cells is essential to reduce the influence of variations in lighting and noise.

The HOG method preserves the local edge or gradient patterns within cells, which enhances its robustness against local geometric and photometric variations. HOG does not require on prior knowledge of leaf anatomy for leaf categorization. It does not extract information from conventional botanical parameters, such as the leaf’s length-width ratio or the number of lobes. Instead, HOG focuses on capturing the local gradient characteristics, making it applicable for leaf classification without explicit botanical feature engineering.

The LBP is another widely-used feature extraction method in texture analysis, valued for its computational simplicity and effectiveness in texture classification^33^. Prior to applying LBP, the images are pre-processed by converting them to grayscale. The LBP operator calculates the difference in gray levels between a central pixel and its neighboring pixels within a defined region. If we denote the gray value of a pixel as *I*(*x*, *y*), the LBP value of that pixel is computed as a decimal using the following formula (10):

10 $$\begin{aligned} L(x,y)=\sum _{i = 0}^{K - 1} f[(I_i (x,y)-I(x,y))] \times 2^i, \end{aligned}$$

where

$$\begin{aligned} f[(I_i (x,y)-I(x,y))]= {\left\{ \begin{array}{ll} 1, &{} \quad \text {if } ~~ I_i (x,y)-I(x,y) \ge 0\\ 0, &{} \quad \text {if } ~~ I_i (x,y)-I(x,y)<0),\\ \end{array}\right. } \end{aligned}$$

and *K* is the number of neighbor pixels around the center one.

It is important to note that HOG may exhibit limited performance when applied to images with noisy edges. In such cases, LBP serves as a potential alternative. LBP features have been extensively employed in diverse applications, demonstrating promising performance in tasks like face recognition. LBP is particularly valuable due to its ability to classify images with respect to their invariance to monotonic gray level changes and its high computational efficiency.

To utilize both HOG and LBP effectively, the feature vectors extracted from training and testing images using these methods were concatenated. This concatenation resulted in a new feature vector that combined the information captured by both techniques. The resulting feature vector was then employed as the final feature representation during the classification stage. This approach helps leverage the strengths of both HOG and LBP, enhancing the overall performance of the classification algorithm. Furthermore, we present visualizations using histograms of leaf data captured through two distinct methods, namely HOG and LBP. Specifically, these histograms showcase the leaf data of four randomly chosen plant specimens, namely *Ocimum gratissimum*, *Combretum indicum*, *Thevetia peruviana*, and *Senna alata*.

**Figure 3:**
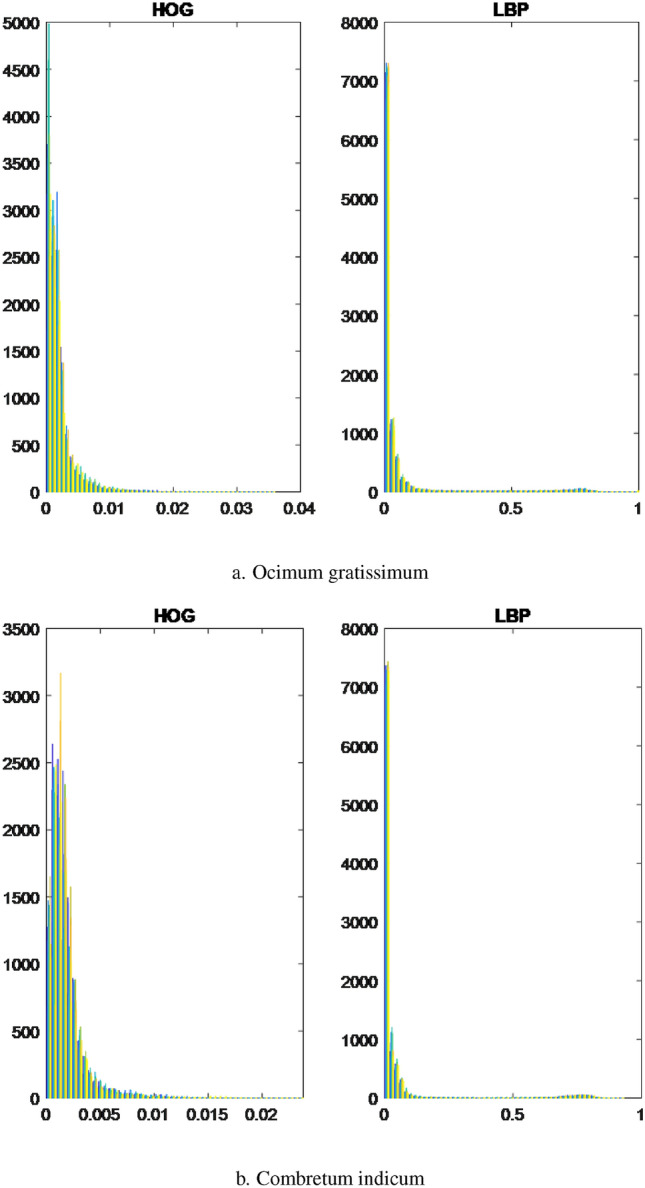
The histograms of the leaves are based on characteristic extraction using HOG and LBP methods.

**Figure 4:**
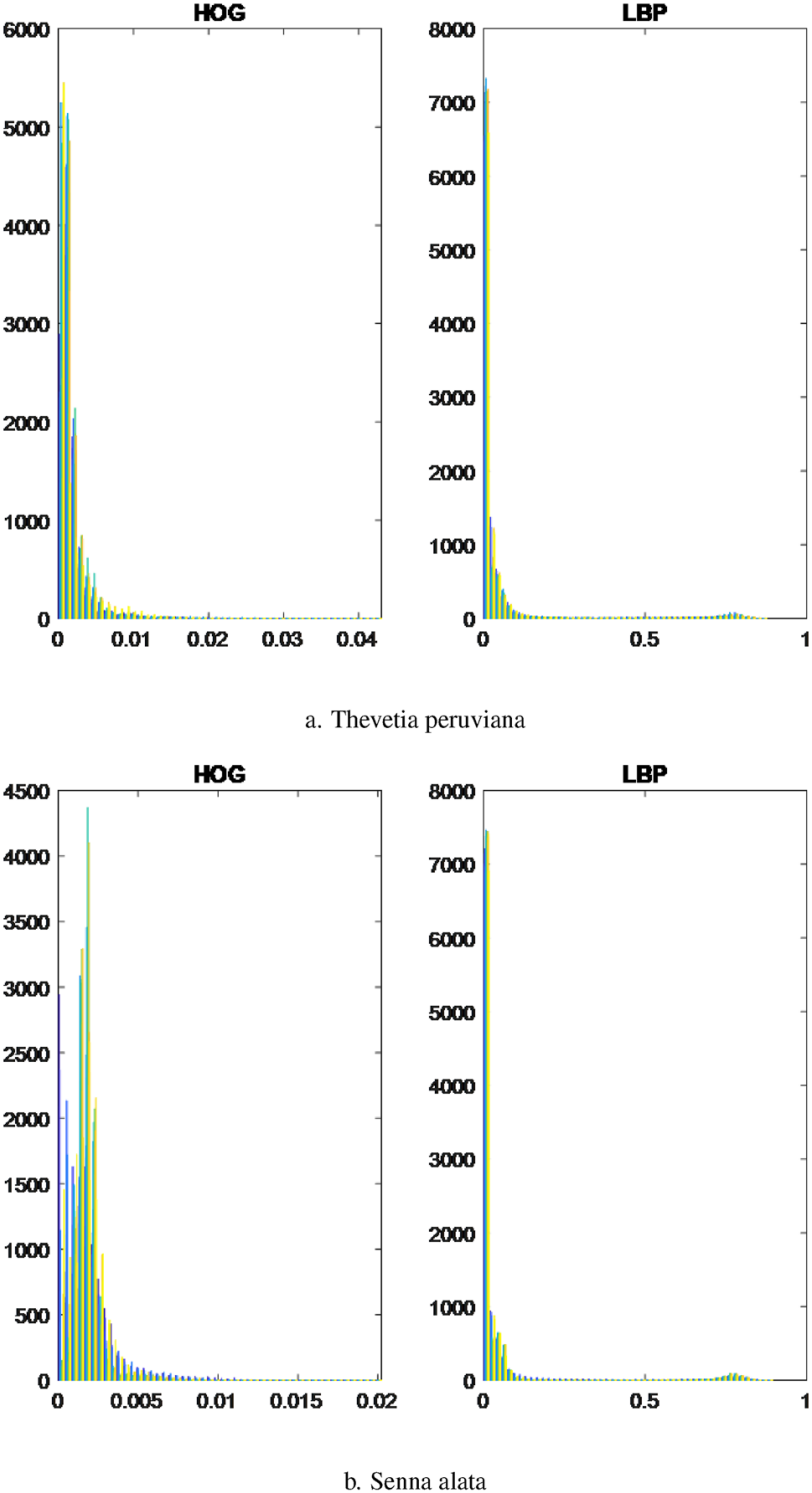
The histograms of the leaves are based on characteristic extraction using HOG and LBP methods.

Based on the observations from Figs. 3a,b and  4a,b, we observe that the differences between the leaves, as extracted using LBP, appear to be quite similar, which presents challenges in classification. In contrast, the differences between the leaves are visually discernible when using HOG for feature extraction. However, the approximation of the leaf data using a normal distribution is relatively restrictive, necessitating a more accurate approximation of the probability distribution form for the leaf data.

Considering the aforementioned situation, it would be more suitable to approximate the data using a single skewed probability distribution, as opposed to a mixture of normal distributions, skewed probability distributions, or a combination of normal and skewed distributions. In the case of mixed probability distributions, it becomes crucial to accurately estimate the parameters of the component probability distributions. The parametric estimation of these distributions relies on the stability of the selected parameters, taking into account the characteristics of the sub-dataset.

In our dataset, we have collected images of various plant species, where factors such as care, soils, and leaf age contribute to variations in leaf size. The probability distributions of these data samples exhibit approximate similarity.

**Figure 5:**
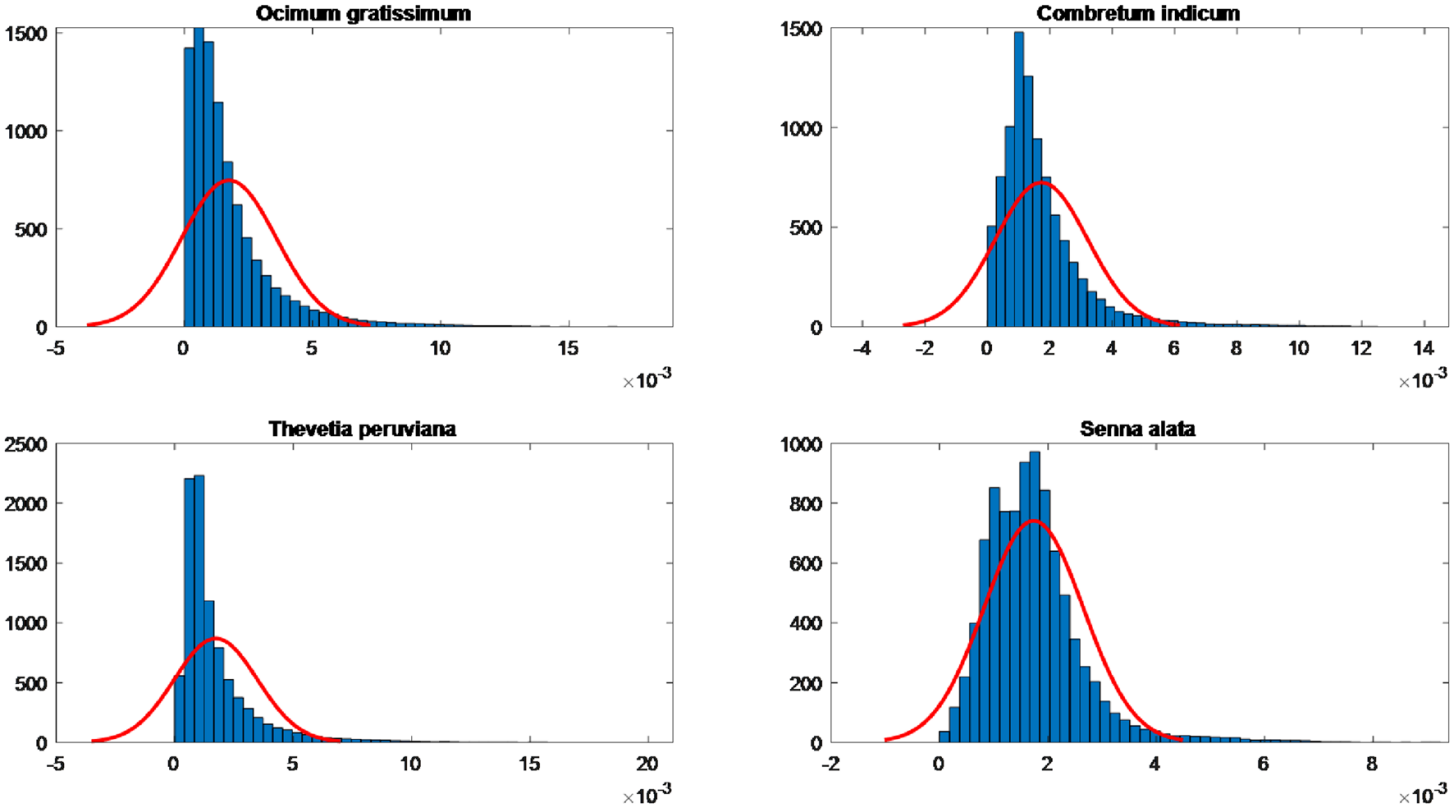
Approximately normal distribution of data corresponding leaves four categories based on the average method.

For data pre-processing, we apply the HOG method to extract leaves from collected data. We propose two methods to pre-process the data: the average and the fuzzy methods. For the averaging method, we sorted the data in ascending order. Specifically, at each position, we calculate the average of all values in each position. Then, the graph of the aggregated data set from each leaf data type is shown in Fig. 5. Furthermore, the leaf data sets extracted by the HOG method, in which mean and standard deviation parameters are approximately the same, are shown in Table 3.

**Table 3:** The parameters of the data set of leaves were extracted by the HOG method.

Types of leaves	Parameters	
	Mean	Standard deviation
*Ocimum gratissimum*	0.0017	0.0018
*Combretum indicum*	0.0017	0.0015
*Thevetia peruviana*	0.0017	0.0018
*Senna alata*	0.0017	0.0009

Given that the datasets exhibit the same mean and standard deviation, estimating the data using the same probability distribution will yield identical results. Therefore, it becomes essential to estimate the parameters using Bayesian statistics, as outlined in Algorithm 2. This approach ensures parameter stability by adjusting them based on the available data.

The estimation of parameters from the probability distribution is performed using the maximum a posteriori (MAP) approach, considering loss functions such as the mean for quadratic loss, the median for absolute loss, or the mode for 0–1 loss. However, in many cases, it is desirable to estimate specific values, including potentially large values. To address this, the parameters are treated as fuzzy numbers, allowing for a more flexible representation. The characterization function of the fuzzy numbers can be determined based on the adjusted probability density function corresponding to those parameters.

In the fuzzy number method, the data is sorted in ascending order, and instead of calculating averages, range values from the smallest to the largest are computed at each position. As a result, the data is transformed into fuzzy intervals, where the estimated parameters also become fuzzy numbers. Details of relevant knowledge about fuzzy numbers are presented in the next section.

## Fuzzy numbers and the fuzzy Kullback–Leibler divergence

### Fuzzy numbers and characterizing functions

#### Definition 1^26^

A fuzzy number $x^*$ is determined by its characterizing function $\xi (\cdot )$, which is a real function of one real variable *x* following several requirements:

- $$\xi :{\mathscr {R}} \rightarrow [0;1].$$
- $\forall \delta \in (0;1]$ the so-called $\delta$-cut $C_\delta (x^* )=x \in {\mathscr {R}}: \xi (x) \ge 0$ is a finite union of compact intervals, $[a_{\delta ,i};b_{\delta ,i} ]$, i.e. $C_\delta (x^* )=\cup _{j=1}^k [a_{\delta ,j};b_{\delta ,j} ] \ne 0.$
- The support of $\xi (.)$, defined by $supp[\xi (.)]=\{x \in {\mathscr {R}}: \xi (x)>0\}$, is bounded.

The set of all fuzzy numbers is denoted by ${\mathscr {F}}({\mathscr {R}})$.

#### Definition 2^26^

A fuzzy number is called a fuzzy interval if all its $\delta$-cuts are non-empty closed bounded intervals. The set of all fuzzy intervals is denoted by ${\mathscr {F}}_I ({\mathscr {R}})$.

### Mathematical operations for fuzzy quantities

Let $x_1^*$ and $x_2^*$ be two fuzzy numbers that have the corresponding characterizing functions $\xi _1 (.)$ and $\xi _2 (.)$. For fuzzy intervals $x_1^*$ and $x_2^*$, the generalized addition is defined using $\delta$-cuts, $C_\delta (x_1^* )=[a_{\delta ,1};b_{\delta ,1}]$ and $C_\delta (x_2^* )=[a_{\delta ,2};b_{\delta ,2}],\forall \delta \in [0;1]$ then the $\delta$-cut of the fuzzy sum $x_1^* \oplus x_2^*$ is given by (11)^26^:

11 $$\begin{aligned} C_\delta (x_1^* \oplus x_2^* )=\left[ a_{\delta ,1}+a_{\delta ,2};b_{\delta ,1}+b_{\delta ,2} \right] , \forall \delta \in (0;1]. \end{aligned}$$

The characterizing function of $x_1^* \oplus x_2^*$ is determined by Lemma 1.

#### Lemma 1^26^

The characterizing function $\xi (\cdot )$ of a fuzzy number $x^*$ holds following condition:

12 $$\begin{aligned} \xi (x)=\max \{ \delta \times I_{C_\delta (x^* )}(x): \delta \in [0;1] \}, \forall x \in {\mathscr {R}}. \end{aligned}$$

The generalizing product $x_1^* \otimes x_2^*$ of two fuzzy numbers with corresponding characterizing functions $\xi _1 (\cdot )$ and $\xi _2 (\cdot )$ is defined by formula (13)^26^:

13 $$\begin{aligned} \xi _{x_1^* \otimes x_2^*} (x)= \sup \{ \min \{ \xi _1 (x_1 ),\xi _2 (x_2) \}: x_1 \times x_2= x \},\forall x \in {\mathscr {R}}. \end{aligned}$$

The $\delta$-cuts of the product $x_1^* \otimes x_2^*$ are calculated by (14)^26^:

14 $$\begin{aligned} C_\delta (x_1^* \otimes x_2^* )= \left[ \left( \min _{(x_1,x_2 ) \in C_\delta (x_1^* ) \times C_\delta (x_2^* )} x_1 \times x_2 \right) ; \left( \max _{ (x_1,x_2 ) \in C_\delta (x_1^*) \times C_\delta (x_2^*)} x_1 \times x_2 \right) \right] ,\forall \delta \in (0;1]. \end{aligned}$$

#### Proposition 1^26^

Let $x_1^*,x_2^*,\ldots ,x_n^*$ be fuzzy intervals with $\delta$-cuts: $C_\delta (x_i^* )=[a_{\delta ,i};b_{\delta ,i} ]$, then the fuzzy arithmetic mean: ${\overline{x}}^*=\frac{1}{n} \oplus _{i=1}^n x_i^*$ is a fuzzy interval, and $\delta$-cut determined by (15):

15 $$\begin{aligned} C_\delta ({\overline{x}}^* )= \left[ \frac{1}{n} \sum _{i=1}^n a_{\delta ,i}; \frac{1}{n} \sum _{i=1}^n b_{\delta ,i} \right] , \forall \delta \in (0;1]. \end{aligned}$$

### Fuzzy correlation

Assume the characterizing function $\xi (\cdot )$ of the fuzzy number $x^*$ is described in formula (16)^34^:

16 $$\begin{aligned} \xi _{(x^*)}(t)= {\left\{ \begin{array}{ll} 0, &{}\quad if~~ t<t_l^0,\\ f_{x^*} (t), &{}\quad if~~ t_l^0 \le t< t_l^1,\\ 1,&{}\quad if~~ t_l^1 \le t \le t_r^1,\\ g_{x^*} (t),&{} \quad if~~ t_r^1<t \le t_r^0,\\ 0,&{} \quad if~~ t_r^0<t.\\ \end{array}\right. } \end{aligned}$$

Where the functions $f_{x^*}(.)$ and $g_{x^*}(.)$ are referred as the left and the right sides of $x^*$, respectively. Additionally, the formula (17)^34^ determines the lower and upper expected values of the fuzzy number $x^*$ in the similar manner.

17 $$\begin{aligned} {\underline{E}} (x^* )=t_l^1- \int _{t_l^0}^{t_l^1} f_{x^*} (t) dt, {\overline{E}}(x^* )=t_r^1+\int _{t_r^1}^{t_r^0} g_{x^* } (t) dt. \end{aligned}$$

#### Definition 3^34^

For fuzzy numbers $x^*$ and $y^*$, we define (18) and (19) as the correlation formula and the correlation coefficient of fuzzy numbers $x^*$ and $y^*$.

18 $$\begin{aligned} C(x^*,y^* )= & {} {\underline{E}}(x^* ) {\underline{E}} (y^* )+{\overline{E}} (x^* ) {\overline{E}} (y^* ),\end{aligned}$$

19 $$\begin{aligned} \rho (x^*,y^* )= & {} \frac{C(x^*,y^* )}{\sqrt{C(x^*,x^* )C(y^*,y^* )}}. \end{aligned}$$

The equations in Eqs. (16), (17), (18), (19) have been formulated as generalized forms to handle fuzzy numbers, accommodating not only Gaussian fuzzy numbers but also a wide range of other fuzzy number types. This versatility arises from two main factors. Firstly, the defining function, denoted as $xi_{(x^*)}(t)=1$, is established over an interval $[t_l^1; t_r^1]$ rather than at a single point, ensuring its applicability to fuzzy numbers with diverse shapes and characteristics. Secondly, the left and right functions of the fuzzy number, represented as $f_{x^{*}}(.)$ and $g_{x^{*}}(.)$ respectively, need not be identical, thereby allowing for more flexibility in dealing with different fuzzy number representations. Consequently, these equations offer a robust framework suitable for handling a broad spectrum of fuzzy numbers.

### The fuzzy extended Kullback–Leibler divergence

The estimation of parameters for the component probability distributions within a mixture of probability distributions was performed using Algorithm 2, resulting in the representation of these parameters as probability distributions. To facilitate a more generalized treatment of the parameters, we propose considering them as fuzzy numbers, achieved by adjusting the probability density function to the characterizing function. This adjustment ensures that the maximum value of the probability density function is 1. Specifically, we introduce the adjusted probability density function, denoted as ${\tilde{f}}(\theta )$, which is obtained by dividing the probability density function $f(\theta )$ by the value of the probability density function at the mode, as defined by the formula (20):

20 $$\begin{aligned} \tilde{f}(\theta )= \frac{f(\theta )}{f(\theta _{Mode})}. \end{aligned}$$

Then, the values $\tilde{\theta }$ is the adjusted probability density function $\tilde{f}(\tilde{\theta } ) \ge \delta$ for each level $\delta$-cut, with $0<\delta \le 1$. Since the probability density function follows a single probability distribution, the parameter defined an interval $\tilde{\theta } \in (\tilde{\theta }^l ;\tilde{\theta }^r), \tilde{\theta }^l \le \tilde{\theta }^r$.

When dealing with numerical data represented as fuzzy numbers, the parameters of the fuzzy probability density function also become fuzzy numbers. However, it is important to distinguish between probability distributions with fuzzy interval parameters and fuzzy interval data. In this context, this paper proposes a theorem regarding the fuzzy extended Kullback–Leibler divergence to address the aforementioned challenges and provide a solution. This theorem introduces a novel approach for evaluating the divergence between fuzzy probability distributions, contributing to the advancement of fuzzy inference methods.

#### Theorem 1

Assume *f* and *g* are probability density functions, with the parameters of each probability density function $\theta _{f1}^*,\theta _{f2}^*,\ldots ,\theta _{fk}^*,\theta _{g1}^*,\theta _{g2}^*,\ldots ,\theta _{gh}^*$ are the fuzzy numbers. Then, the fuzzy extended Kullback–Leibler divergence $KLD^* (f||g)$ between two probability density functions $f(x|\theta _{f1}^*,\theta _{f2}^*,\ldots ,\theta _{fk}^*)$ and $g(x|\theta _{g1}^*,\theta _{g2}^*,\ldots ,\theta _{gh}^*)$ is also a fuzzy number, determined by the formula (21):

21 $$\begin{aligned} KLD^* (f||g)=\int _M^* f \left( x|\theta _{f1}^*,\theta _{f2}^*,\ldots ,\theta _{fk}^* \right) \times \log \frac{f \left( x| \theta _{f1}^*,\theta _{f2}^*,\ldots ,\theta _{fk}^*\right) }{g\left( x|\theta _{g1}^*,\theta _{g2}^*,\ldots ,\theta _{gh}^*\right) } dx. \end{aligned}$$

#### Proof

The fuzzy extended Kullback–Leibler divergence $KLD^* (f||g)$ is the integral of the variable *x*, which depends on the fuzzy parameters. Therefore, when the value of the parameter changes, the integration function and the integral result also changes.

On the other hand, the value of the Kullback–Leibler divergence *KLD*(*f*||*g*) always non-negative and bounded^35^. There are the existing minimum and maximum values that measure the difference between two probability distributions.

Specifically, for each $\theta$-cuts of the parameters are $C_\delta (\theta _j^* )=[\theta _j^l;\theta _j^r ], \forall \delta \in (0;1]$, the fuzzy extended Kullback–Leibler divergence $KLD^* (f||g)$ is calculated by (22):

22 $$\begin{aligned} C_\delta [KLD^* (f||g )] = \left[ \min _{\theta _j \in [ \theta _j^l; \theta _j^r ] } fg; \max _{ \theta _j \in [\theta _j^l; \theta _j^r]} fg \right] , \end{aligned}$$

with

$$\begin{aligned} fg= & {} \int _M f \left( x|\theta _{f1},\theta _{f2},\ldots ,\theta _{fk} \right) \times \log \frac{f(x|\theta _{f1},\theta _{f2},\ldots ,\theta _{fk})}{g(x|\theta _{g1},\theta _{g2},\ldots ,\theta _{gh})} dx\\\approx & {} \sum _{t=1}^T Prob_f^* ((x_{t-1};x_t]|\theta _{f1},\theta _{f2},\ldots ,\theta _{fk}) \times \log \frac{Prob_f^* ((x_{t-1};x_t]|\theta _{f1},\theta _{f2},\ldots ,\theta _{fk} )}{Prob_g^* ((x_{t-1};x_t]|\theta _{g1},\theta _{g2},\ldots ,\theta _{gh} )}, \end{aligned}$$

and $$x\in M \subseteq (x_0;x_T ], x_0= \min \{ x \} - \varepsilon , \varepsilon > 0; x_T = \max \{ x \}$$. $$\square$$

Through Theorem 1, for each $\delta \in (0;1]$, we have the $\delta$-cuts of $KLD^* (f||g)$ to determine the difference between the probability distributions when the parameter changes. Therefore, by applying this result, we can make inferences in classification problems as follows: finding the parameter provides the most similarity between two probability distributions, through smallest divergence corresponds to the minimum value of $KLD^* (f||g)$; finding the value domain of the difference between two probability distributions with fuzzy parameters; finding the domain of values of the difference between a probability distribution with fuzzy parameters and the data, through the corresponding empirical distribution.

### Applications

The detailed illustrations we selected random 23 herbal species for verifying the proposed model as presented in the Table 4.

**Table 4:** The random selected 23 herbal species.

No.	Vietnamese herbal species	Scientific names	Sample size
1	Ắc ó	*Acanthus integrifolius*	20
2	An xoa	*Helicteres hirsuta* Lour.	17
3	Bầu đất trắng	*Gynura procumbens* Mer.	17
4	Bỏng nổ	*Euphorbiaceae*	18
5	Cây bọ nắm	*Pouzolzia zeylanica*	18
6	Chân chim 8 lá	*Schefflera arboricola*	22
7	Chè vằng	*Jasminum subtriplinerve*	16
8	Dành dành	*Gardenia jasminoides* Ellis.	17
9	Đinh lăng	*Polyscias fruticosa*	16
10	Duối ô rô	*Streblus ilicifolius* (Vidal) Corner.	17
11	Hoa phấn	*Mirabilis jalapa* L.	23
12	Hương nhu trắng	*Ocimum gratissimum*	28
13	Khổ qua rừng	*Momordica charantia*	16
14	Lược vàng	*Callisia fragrans*	17
15	Mào gà hoa trắng	*Celosia argentea* L.	16
16	Mía đỏ	*Cheilocostus speciosus*	20
17	Muồng trâu	*Senna alata*	23
18	Ngũ trảo	Folium *Viticis negundo*	16
19	Sâm cau lá lớn	*Curculigo capitulata*	18
20	Sử quân tử	*Combretum indicum*	24
21	Thông thiên	*Cascabela thevetia*	23
22	Trang trắng	*Psychotria reevesii* Wall.	20
23	Xảo tam phân	*Paramignya trimera*	18

The selected herbals were classified into four types based on their actual distributions. The first type corresponds to probability density functions with a single skewed peak and a slight difference between the lower and upper bounds. The second type consists of probability density functions with a single balanced peak and a higher difference between the upper and lower bounds. The third type exhibits an inverse form of the probability density function with a small difference between the upper and lower bounds. The fourth type demonstrates an inverse form of the probability density function with a significant difference between the lower and upper bounds.

For each leaf type, we conducted an analysis using both the HOG and LBP methods. The results are presented on the left and right sides of the respective figures. The upper bound is represented by the color orange, while the lower bound is depicted in blue for each collected dataset. Through the implementation of the HOG method, distinct differences were observed among the leaves of the selected herbals. On the other hand, the leaves extracted using the LBP method displayed minimal variations across all herbals, indicating a high degree of similarity.

**Figure 6:**
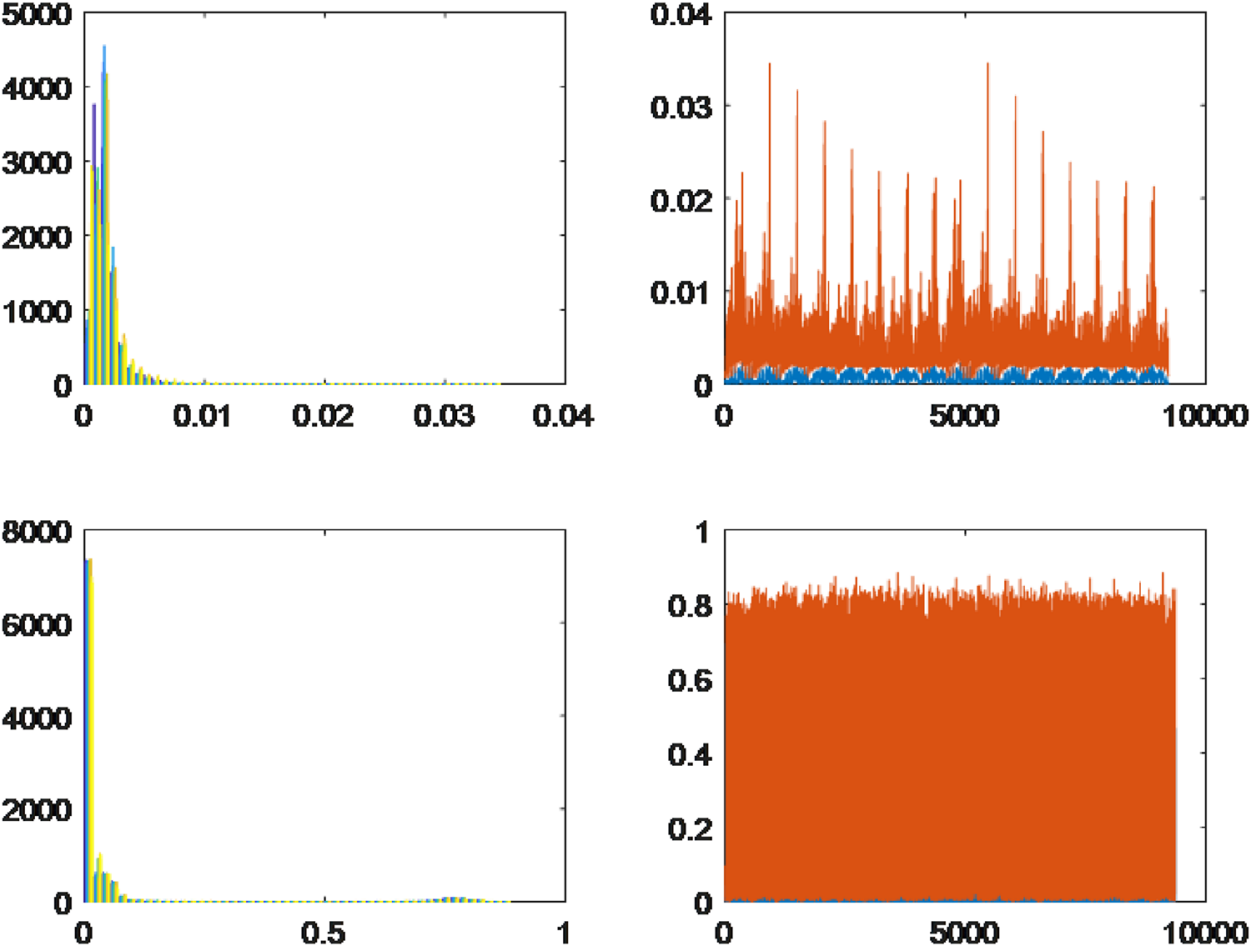
Type 1. *Acanthus integrifolius*.

**Figure 7:**
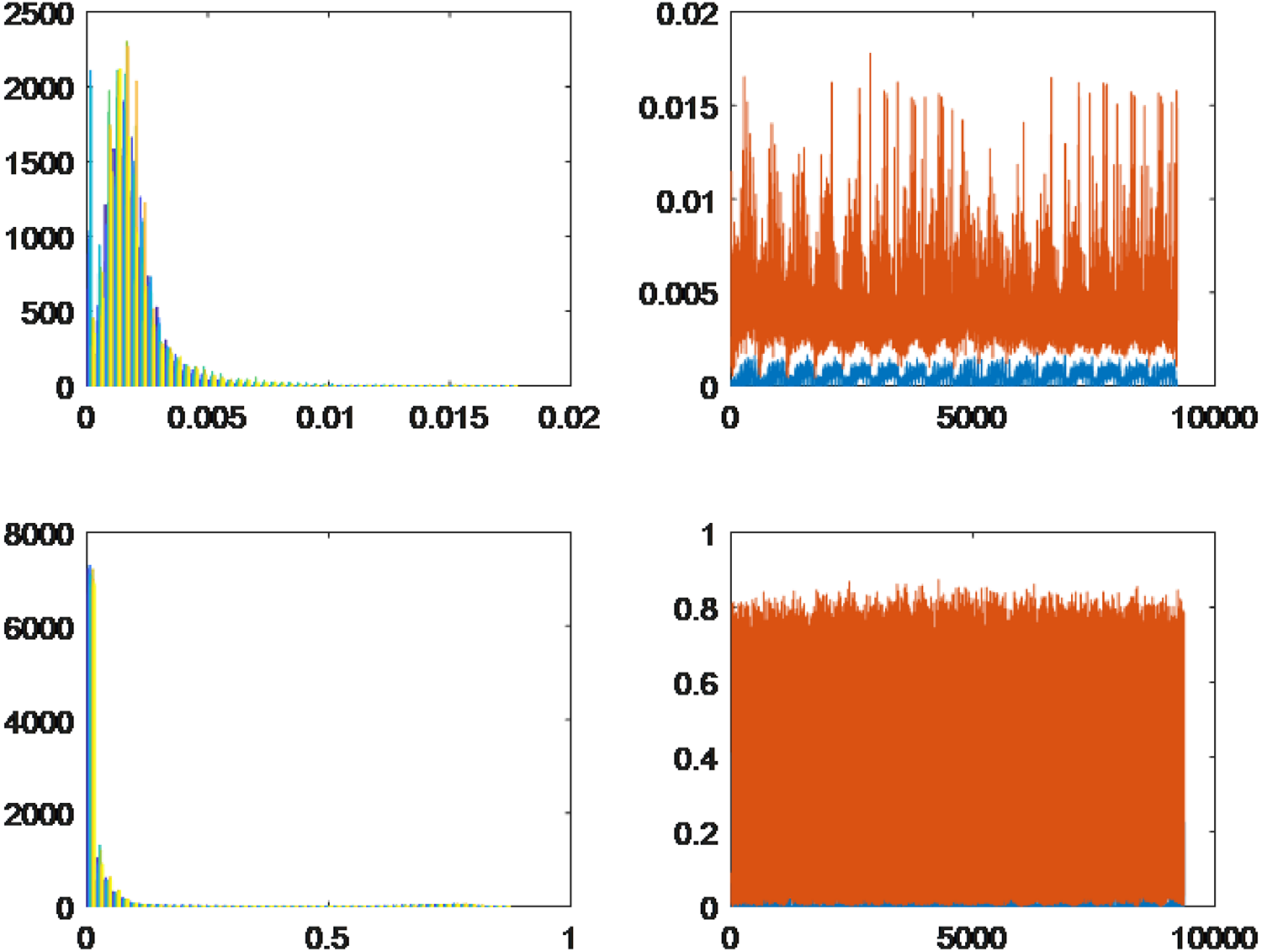
Type 2. *Helicteres hirsuta* Lour.

**Figure 8:**
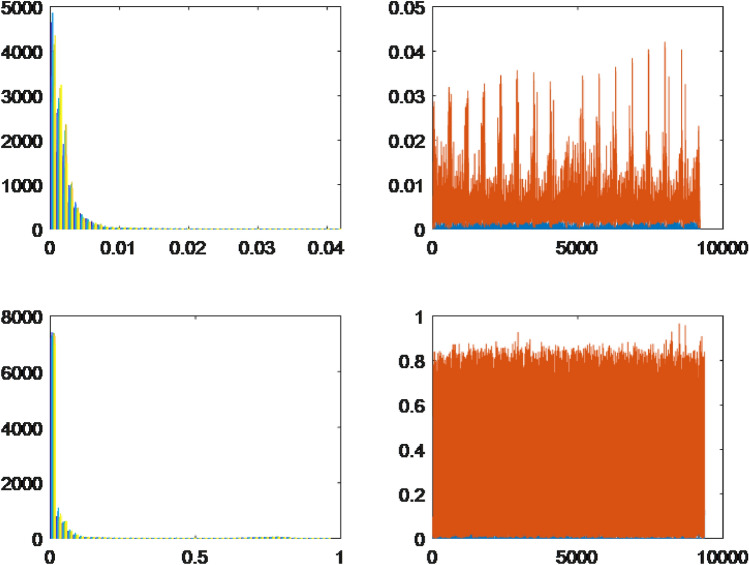
Type 3. *Euphorbiaceae*.

**Figure 9:**
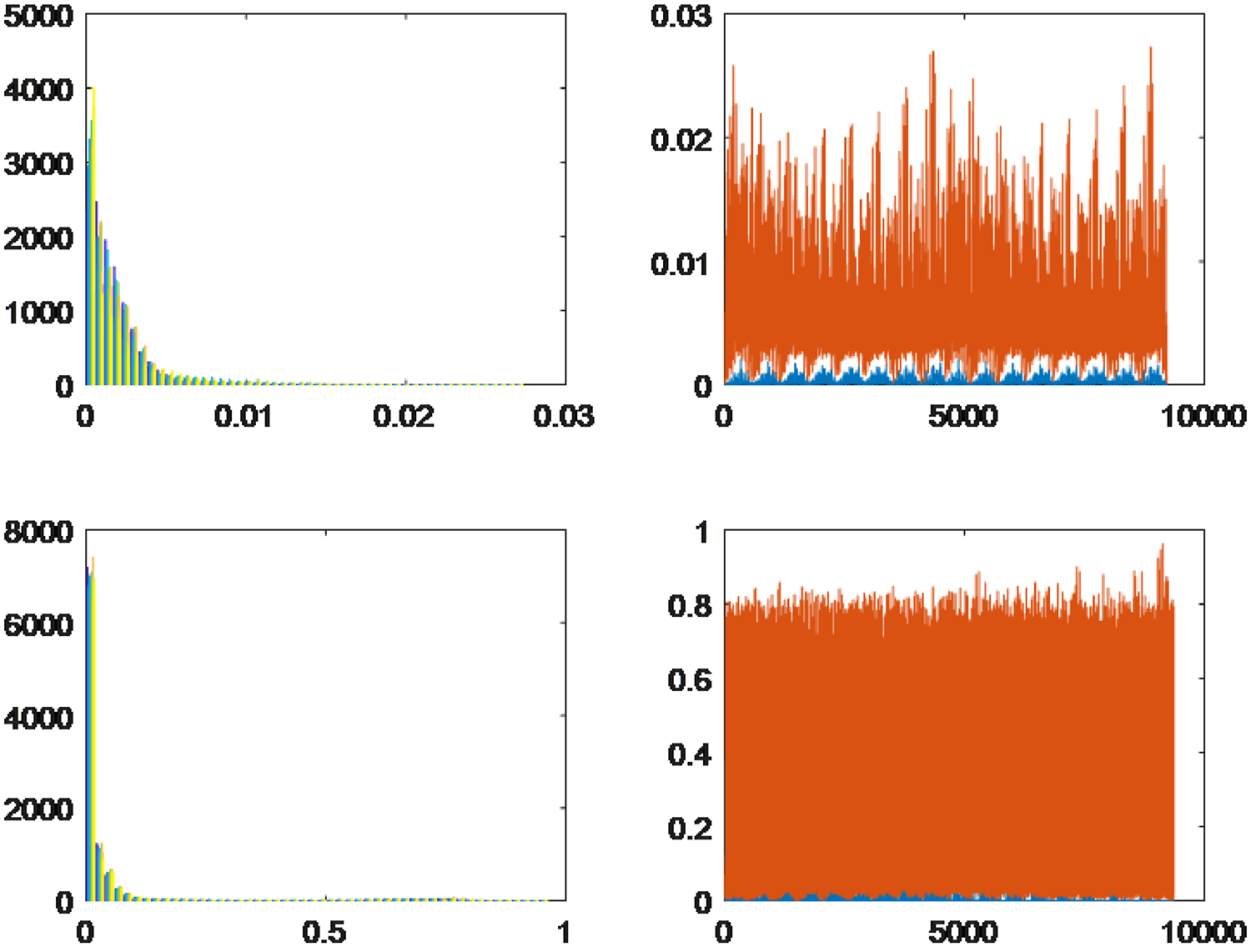
Type 4. Pouzolzia zeylanica.

Type 1.: The first probability density function type has one peak, skewed, and a slight difference between the lower and upper bounds. The results of *Acanthus integrifolius* is illustrated in Fig. 6. The results are similar for trees *Gynura procumbens* Merr., *Jasminum subtriplinerve*, *Streblus ilicifolius* (Vidal) Corner., *Mirabilis jalapa* L., *Paramignya trimera*.
Type 2.: The second type has a single peak, balanced with a higher difference between the upper and lower bounds. The results of *Helicteres hirsuta* Lour. is illustrated in Fig. 7. The results are similar for trees *Senna alata* and *Combretum indicum*.
Type 3.: The third type has the reduced form and a small difference between the upper and lower limits. The results of *Euphorbiaceae* is illustrated in Fig. 8. The results are similar for trees *Momordica charantia* and *Curculigo capitulata*.
Type 4.: The fourth type has the reduced form of the probability density function and much difference between the lower and upper bounds. The results of *Pouzolzia zeylanica* is illustrated in Fig. 9. The results are similar for trees Schefflera arboricola, *Gardenia jasminoides* Ellis., *Polyscias fruticosa*, *Ocimum gratissimum*, *Callisia fragrans*, *Celosia argentea* L., *Cheilocostus speciosus*, Folium *Viticis negundo*, *Cascabela thevetia* and *Psychotria reevesii* Wall.

**Table 5:** Cost comparison of CNNs and proposed method for leaf recognition on V-Herb database.

Methods accuracy	(%)
Xception	98.86
Inception ResNet	98.86
EfficientNet-B2	98.86
Mobilenet-V1	97.73
Proposed method	98.87

The results presented in Table 5 provide valuable insights into the performance of the method we introduced compared to several established Convolutional Neural Networks (CNNs), namely Xception, Inception ResNet, EfficientNet-B2, and MobileNet-V1. These comparisons are crucial for evaluating the efficacy of our proposed approach and understanding its relative strengths and weaknesses. Upon analyzing the accuracy scores, we observe that our method achieved an accuracy of 98.86%, which is on par with the best-performing CNNs in the comparison. Xception, Inception ResNet, and ResNet achieved the same accuracy of 98.86%, while EfficientNet-B2 and MobileNet-V1 attained accuracies of 97.73% and 98.87%, respectively. This similarity in accuracy scores suggests that our proposed method holds its ground against well-established CNN architectures and demonstrates its competency in handling the V-Herb database.

## Conclusion

The analysis of probability distributions offers valuable insights into understanding the characteristics of a given dataset. Among these distributions, normal distributions are commonly observed in scientific and engineering domains. Fuzzy sets provide a powerful approach for modeling normal distributions and yielding more accurate results compared to other methods, thanks to their capability to capture nonlinearities in the data^36^. Assumptions made about probability distributions effectively shed light on their behavior and characteristics^37,38^. For instance, when assuming a normal distribution, parameter estimation in Bayesian statistics typically focuses on specific values such as mean, median, or mode, based on the corresponding loss functions to achieve maximum posterior probability. Moreover, each parameter is estimated based on a single specific value. Consequently, it becomes necessary to modify the probability density function of the parameters by considering them as characterizing functions to accommodate fuzzy numerical parameters.

In cases where the parameters are represented as fuzzy numbers, we introduce Theorem 1, which enables the comparison of similarity between probability distributions using the fuzzy extended Kullback–Leibler divergence. This theorem finds application in scenarios such as identifying specific parameter values that minimize the divergence or comparing the differences between two fuzzy parametric probability distributions by comparing their respective $\delta$-cut intervals using extended fuzzy divergences. Prior to determining the difference between the probability distribution and the data, one of the two probability distributions is substituted with the empirical distribution.

By leveraging these concepts and methodologies, we gain valuable insights into probability distributions, their parameters, and their comparisons with empirical data. This enables us to make informed decisions and draw meaningful conclusions in various statistical and modeling applications. While our current research primarily focuses on fuzzy logic, which has been widely adopted and proven effective in various domains such as AI, machine learning, and control systems, we acknowledge that neutrosophic statistics represents a promising field with substantial potential for future studies and applications.

In practical scenarios, it is uncommon for a dataset to strictly adhere to a single probability distribution. More often, datasets exhibit a mixture of probability distributions, where multiple distributions need to be combined to obtain accurate results. Rather than assuming a specific probability distribution beforehand, the appropriate approach involves identifying the underlying component distributions. Understanding the components within a mixture of probability distributions is crucial for accurate interpretation of the results. This process of identifying the component probability distributions within a mixture is essential to avoid confusion and misinterpretation of observations.

Estimating the probability distribution of the data becomes challenging when working with only a subset of informative data, as this can lead to misidentification of the probability distribution. Therefore, careful consideration is necessary to avoid errors in estimating the probability distribution with incomplete data. Our proposed Algorithm 1 determines the single probability distribution that best fits the data based on the extended Kullback–Leibler divergence. However, due to the presence of incomplete data, estimating each parameter of the probability distribution using only a single value can lead to inaccuracies. Hence, it becomes necessary to consider the parameters in the form of probability density functions, employing a Bayesian statistical perspective. This involves selecting the probability density function that best fits the data and following the detailed calculation steps outlined in Algorithm 2 to determine the parameters of the probability distribution.

The disadvantage of a mixture of normal distributions is that it may not always be the correct probability distribution for the data. When the data is better represented using skewed distributions, fewer data points are required and higher precision can be achieved. Therefore, post-testing processes are necessary to ensure that the estimated parameters of the probability distribution or mixture of probability distributions are based on reliable measures such as AIC, BIC, entropy, cross-validation, or hypothesis testing. Among these measures, the Kullback–Leibler Divergence offers several advantages in accurately evaluating the parameters of probability distribution functions. Another limitation in testing the accuracy of fuzzy probability distributions is the consideration of only certain variables, such as expected value, variance, covariance, and entropy, while neglecting others. In Bayesian statistics, parameter estimates mainly involve specific values like mean, median, or mode, determined by the corresponding loss functions to ensure maximum posterior probability. Each parameter is estimated based on a specific value. To address this, it is necessary to modify the probability density function of the parameters using a characterizing function that accounts for fuzzy numerical parameters. In cases where the parameters are represented as fuzzy numbers, we introduce Theorem 1, which enables the comparison of similarity between probability distributions using the fuzzy extended Kullback–Leibler divergence. The applications of Theorem 1 include finding specific parameter values that minimize the divergence and comparing the differences between two fuzzy parametric probability distributions by evaluating the respective $\delta$-cut intervals of the corresponding extended fuzzy divergences. Before determining the difference between the probability distribution and the data, one of the two probability distributions is substituted with the empirical distribution.

It is challenging to determine the true form of a probability distribution when relying on distribution assumptions. In many cases, real-world data exhibits a mixture of probability distributions, further complicating the problem. To address these challenges, we propose a parametric method for estimating fuzzy probability distributions. The novelty of our approach lies in estimating the component probability distributions of the Gaussian Mixture Model. In this context, the mixture of probability distributions is defined as a combination of normal distributions using the EM technique. Bayesian statistics and our proposed fuzzy extended Kullback–Leibler divergence are employed for parameter estimation and assessing the similarity between probability distributions, respectively. To demonstrate the applicability of our research, we present an illustrative case study involving Vietnamese herb leaves. The experimental results showcased in this paper demonstrate the effectiveness of the proposed method for datasets exhibiting similar characteristics.

## Data Availability

The datasets generated and analyzed during the current study are not publicly available due to its proprietary nature but are available from the corresponding author on reasonable request.
